# Supplementary material for: Molecular basis for the catalytic mechanism of human neutral sphingomyelinases 1 (hSMPD2)
Source: Nat Commun. 2023 Nov 27;14:7755. doi: 10.1038/s41467-023-43580-w (PMC10682184; doi:10.1038/s41467-023-43580-w)
Supplement: Supplementary file 1 — Supplementary Information [file 41467_2023_43580_MOESM1_ESM.pdf]

Supplementary Information

Molecular basis for the catalytic mechanism of human neutral sphingomyelinases 1 (hSMPD2)

Jingbo Yi[1, *], Boya Qi[1, *], Jian Yin[1, *], Ruochong Li[1], Xudong Chen[1], Junhan Hu[1], Guohui Li[2], Sensen Zhang[1, #] Yuebin Zhang[2, #],Maojun Yang[1, 3, #]

[1]Ministry of Education Key Laboratory of Protein Science, Tsinghua-Peking Joint Center for Life Sciences, Beijing Advanced Innovation Center for Structural Biology, School of Life Sciences, Tsinghua University, Beijing 100084, China

[2]State Key Laboratory of Molecular Reaction Dynamics, Dalian Institute of Chemical Physics, Chinese Academy of Sciences, Dalian, China

[3]Cryo-EM Facility Center, Southern University of Science & Technology, Shenzhen, China.

*These authors contributed equally to this work.

#To whom correspondence should be addressed:

Sensen Zhang: zhang.ss@phytovent.com

Yuebin Zhang: zhangyb@dicp.ac.cn

Maojun Yang: maojunyang@tsinghua.edu.cn

This PDF file includes:

Supplementary Figs 1-14 and Figure legends

Supplementary Tables 1-2

Supplementary figures and Figure legends

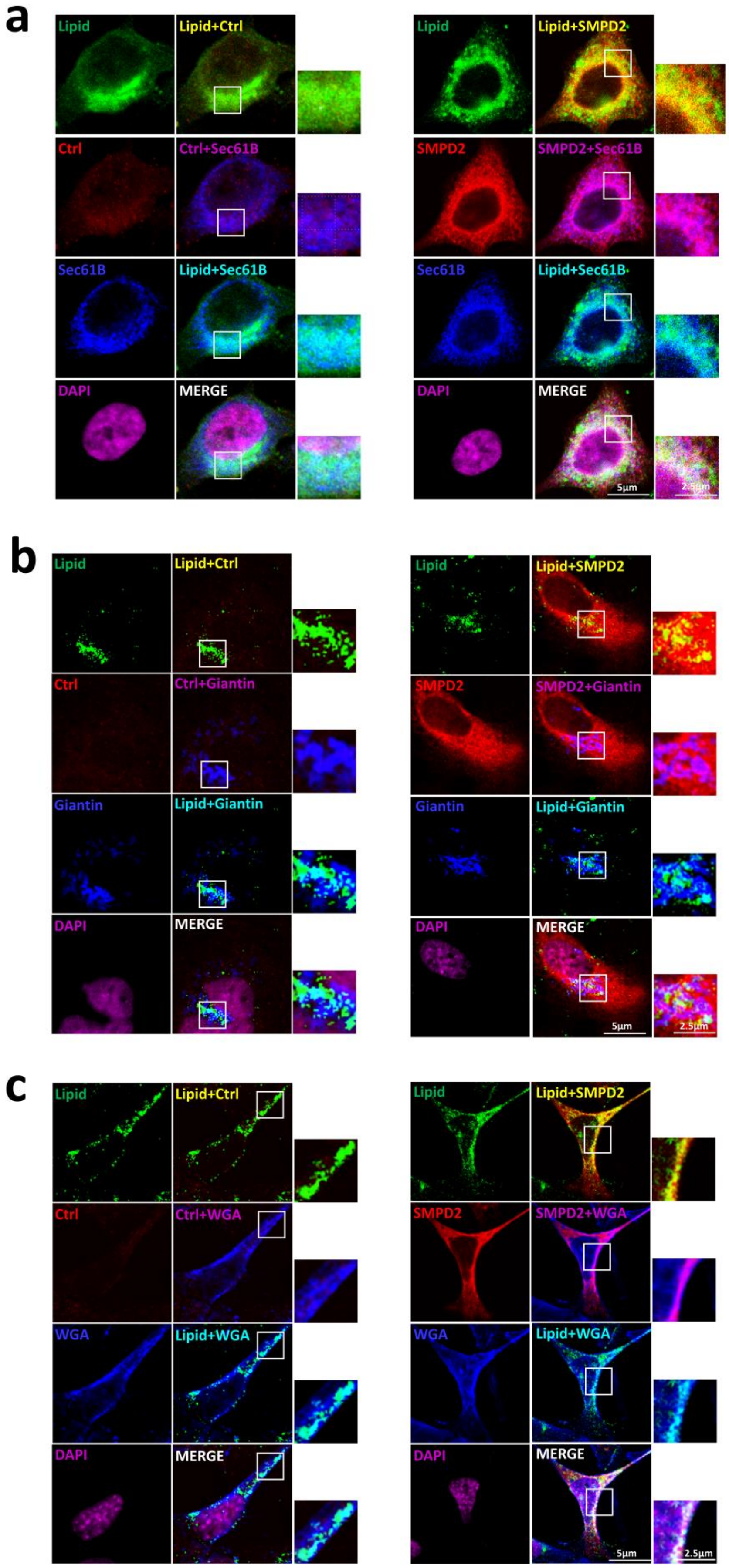

Supplementary Fig.1 Colocalization of the overexpressed hSMPD2 with BODIPY FL labeled sphingomyelin.

a. Immunofluorescence of cells with overexpressed hSMPD2 and Sec61B (ER marker). Left panel (Ctrl); right panel (overexpressed hSMPD2); Scale bar, 2.5 μm.

b. Immunofluorescence of cells with overexpressed hSMPD2 and Giantin (Golgi marker). Left panel (Ctrl); right panel (overexpressed hSMPD2); Scale bar, 2.5 μm.

c. Immunofluorescence of cells with overexpressed hSMPD2. Wheat Germ Agglutinin (WGA) was used to label the plasma membrane. Left panel (Ctrl); right panel (overexpressed hSMPD2); Scale bar, 2.5 μm.

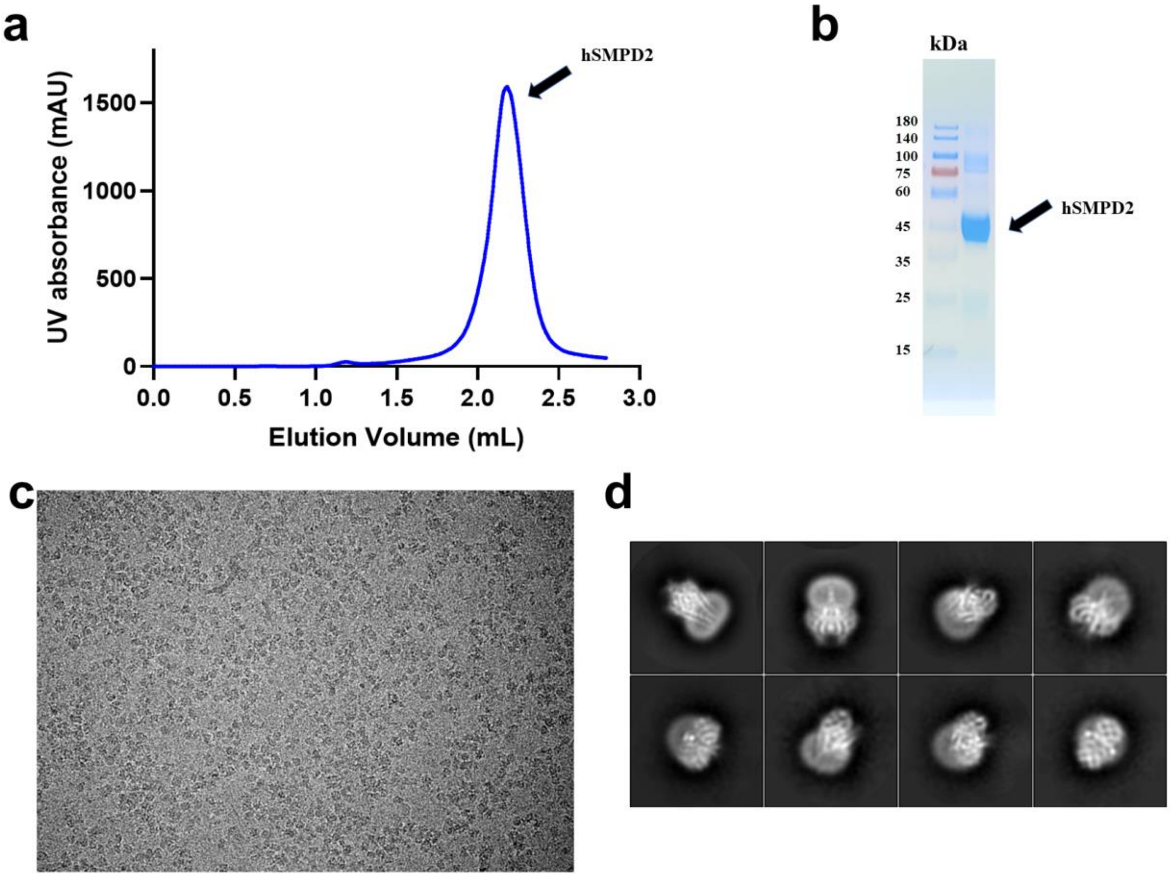

Supplementary Fig.2 Biochemical characterization of hSMPD2.

a. Gel filtration chromatography of hSMPD2.

b. The peak corresponding to the hSMPD2 was subjected to SDS-PAGE and Coomassie blue staining. Source data are provided as a Source Data file.

c. Representative cryo-EM micrograph of hSMPD2 in LMNG buffer.

d. 2D class averages of the hSMPD2 sample in LMNG buffer.

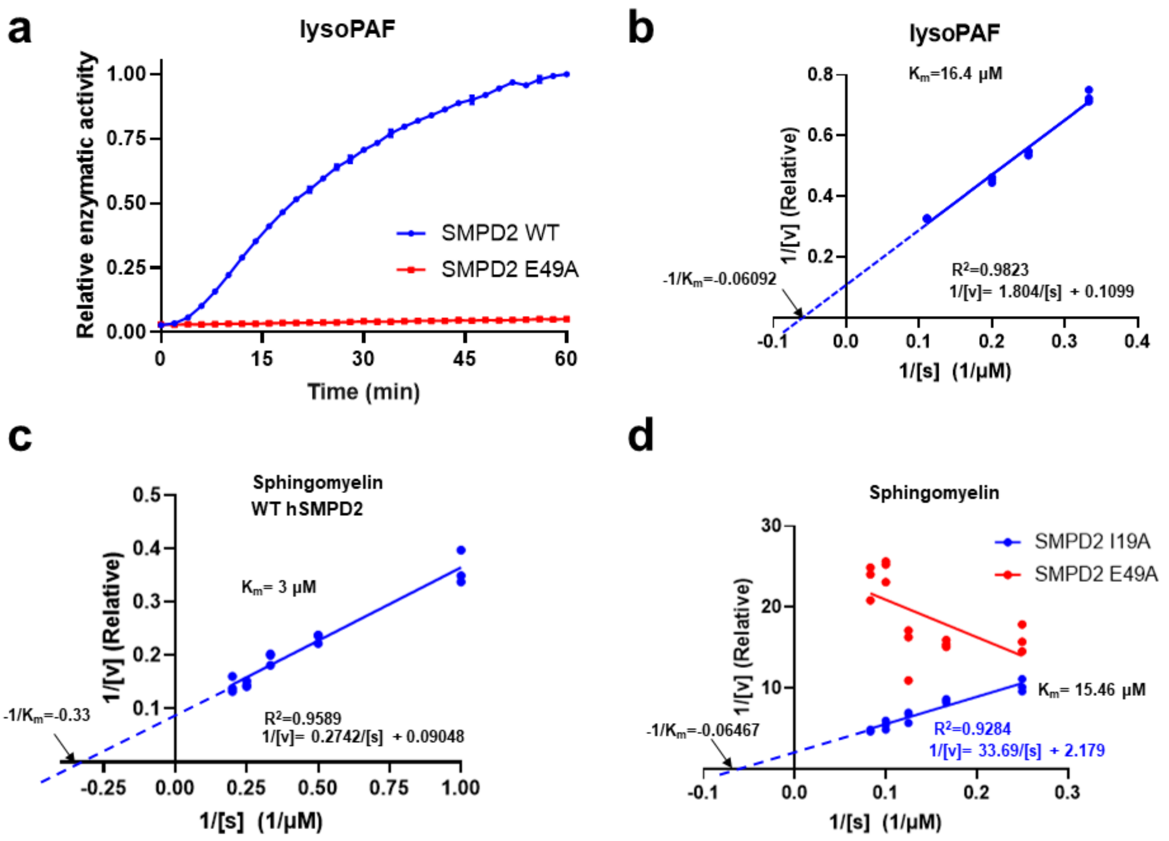

Supplementary Fig.3 Enzymatic kinetic study of hSMPD2 on SM and lyso-PAF.

a. Relative enzyme activity of wild type hSMPD2 and E49A mutants using the Amplex Red Sphingomyelinase Assay Kit. Data shown are mean +/- SEM, *n*=3 biological replicates. Source data are provided as a Source Data file.

b. Enzymatic *Km* value study of WT hSMPD2 by the lyso-PAF substrate, *n*=3 biological replicates. Source data are provided as a Source Data file.

c. Enzymatic *Km* value study of WT hSMPD2 by the SM substrate, *n*=3 biological replicates. Source data are provided as a Source Data file.

d. Enzymatic *Km* value study of hSMPD2 mutants (I19A and E49A) by the SM substrate, *n*=3 biological replicates. Source data are provided as a Source Data file.

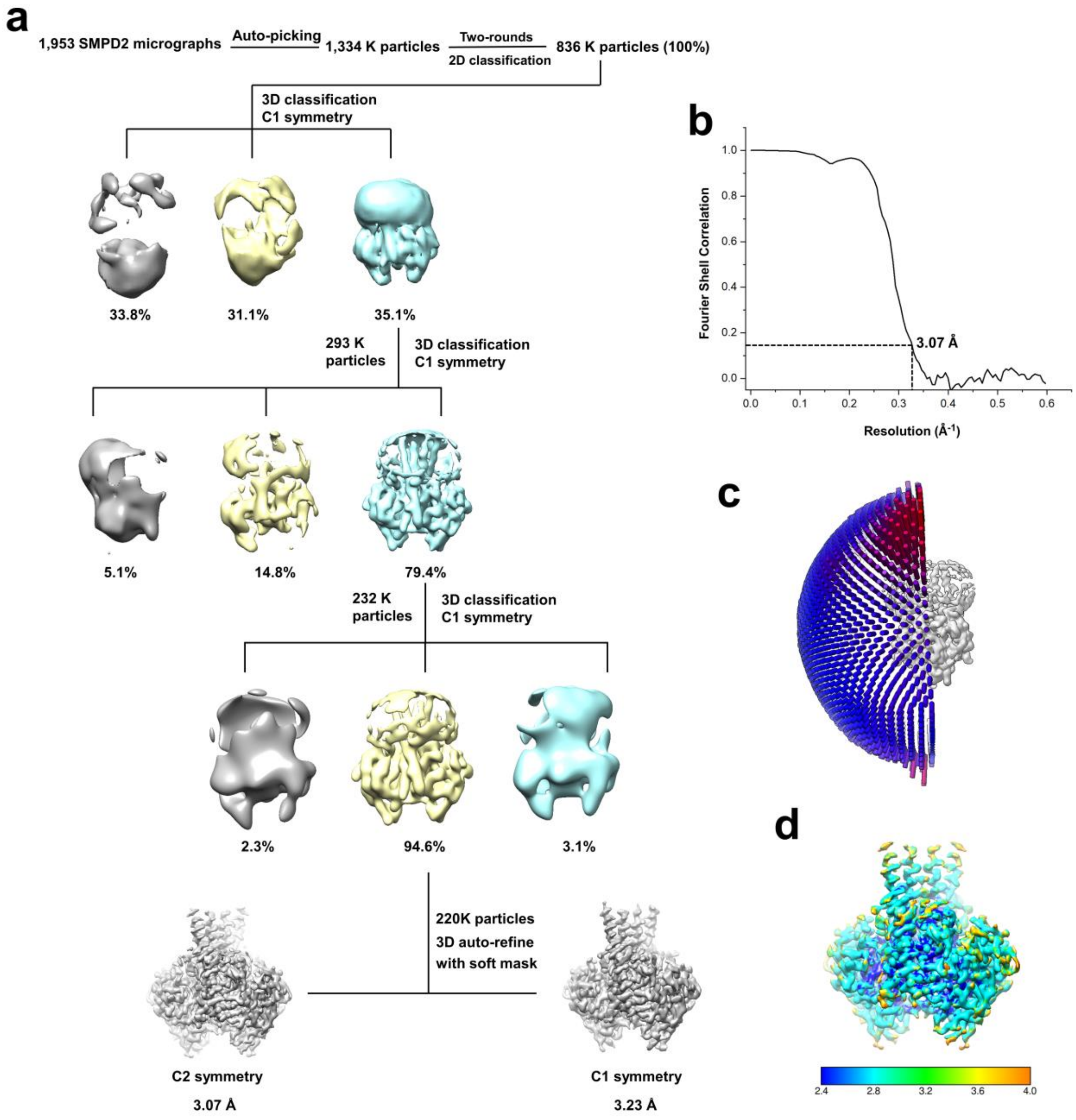

Supplementary Fig.4 Protein purification and structure determination of hSMPD2.

a. The workflow of 2D/3D reconstruction with hSMPD2 cryo-EM data. In brief, 836 k particles were kept after 2D classification, and subjected to three rounds of 3D classification. A final dataset containing 220 k particles were used for high-resolution refinement (see methods for more details).

b. Gold-standard Fourier Shell correlation (FSC) curves of hSMPD2 after 3D refinement. Resolution estimation of hSMPD2 from Relion was based on the criterion of FSC 0.143 cutoff.

c. Angular distribution of the hSMPD2 map final reconstruction.

d. Local resolution map of the hSMPD2 final 3D density map.

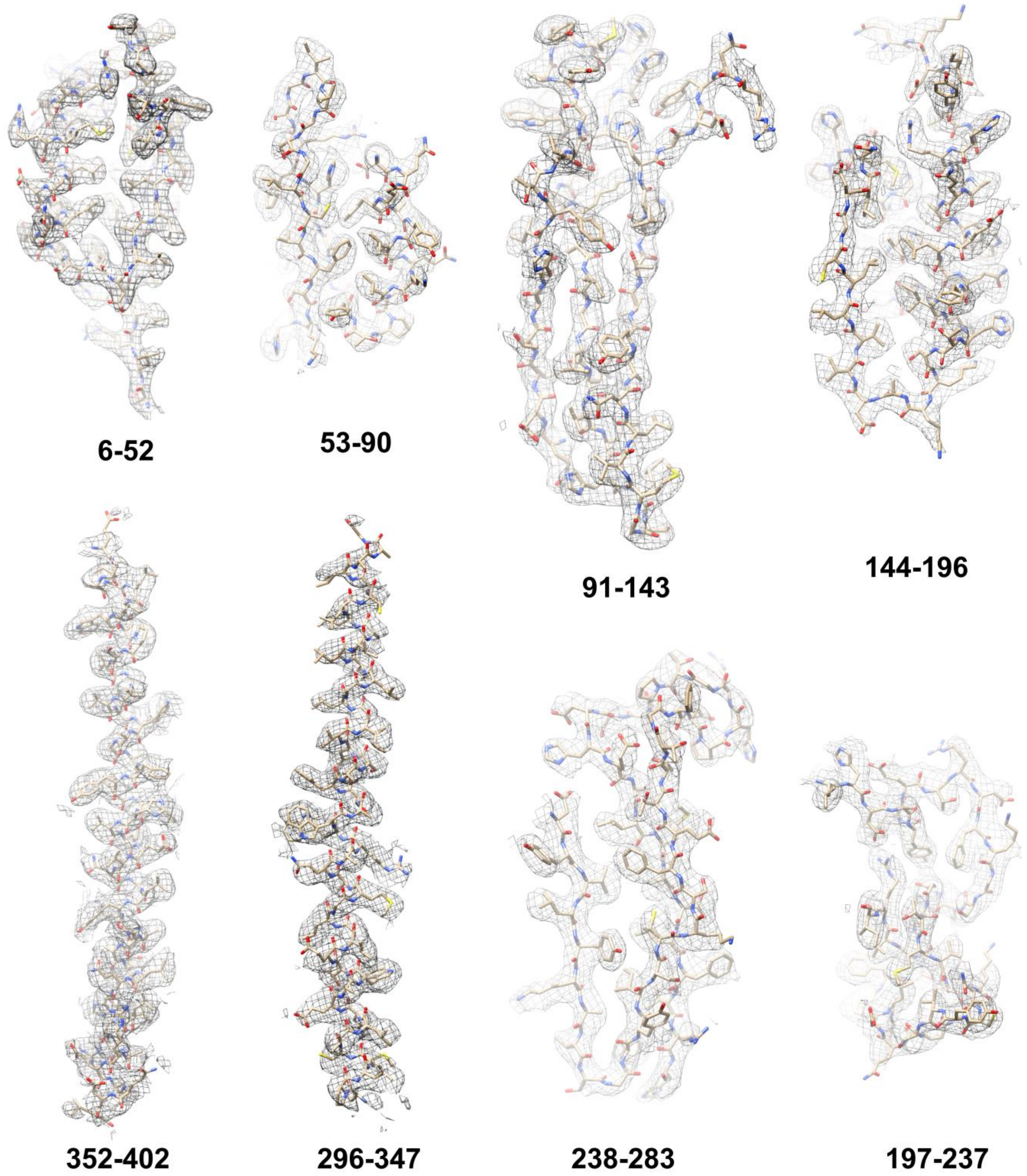

Supplementary Fig.5 Reconstruction and representative cryo-EM densities of hSMPD2

Density maps of representative regions of hSMPD2. Stick style atomic models (gold) were fitted into the cryo-EM density maps (gray mesh). The density maps were contoured at 8.0 σ

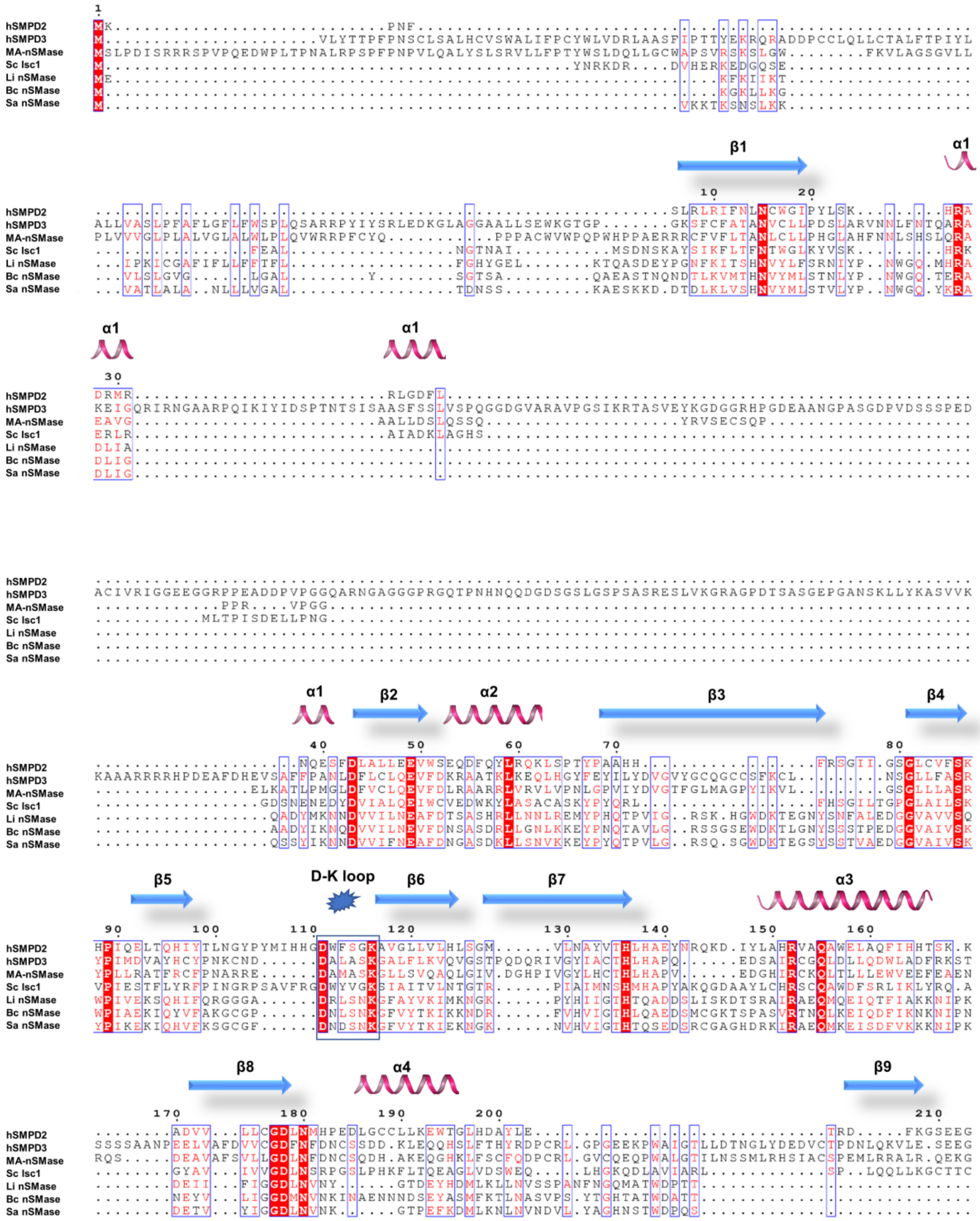
hSMPD2
hSMPD3
MA-nSMase
Sc Isc1
Li nSMase
Bc nSMase
Sa nSMase
β1
α1
α2
β2
β3
β4
β5
D-K loop
β6
β7
α3
β8
α4
β9

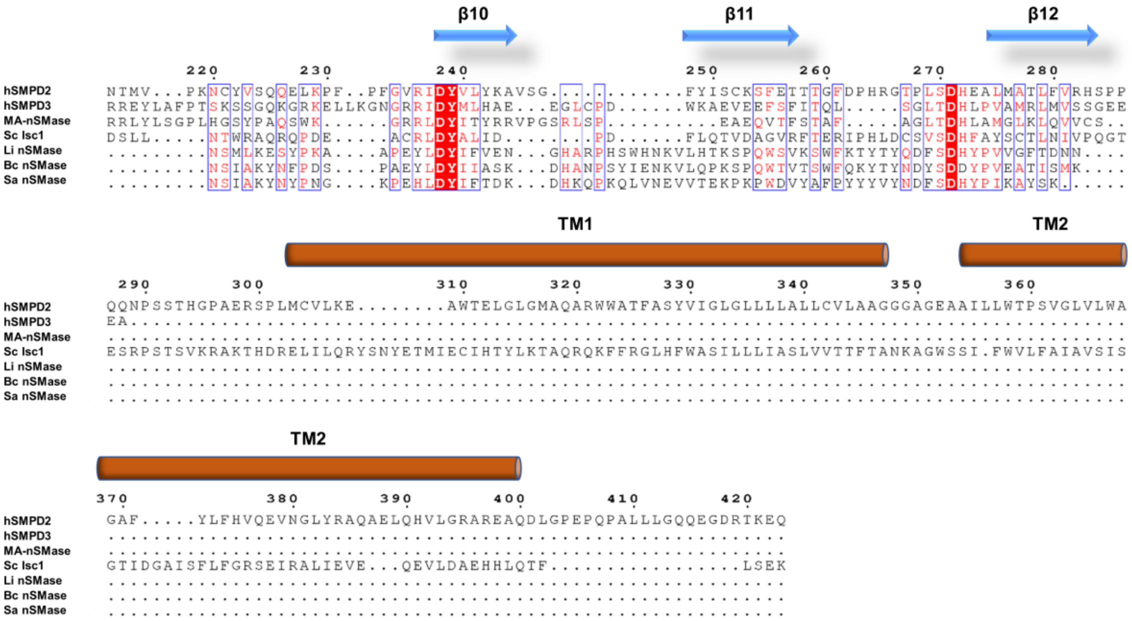

Supplementary Fig.6 Sequence alignment of human SMPD family.

Sequence alignment of hSMPD2 (UniProtKB: O60906), hSMPD3 (UniProtKB: Q9NY59), MA-nSMase (UniProtKB: D6MZJ6), Sc Isc1 (UniProtKB: P40015), Li nSMase (UniProtKB: Q9RLV9), Bc nSMase (UniProtKB: P09599), Sa nSMase (UniProtKB: P09978). Secondary structural elements were indicated as helices, arrows, and cylinders.

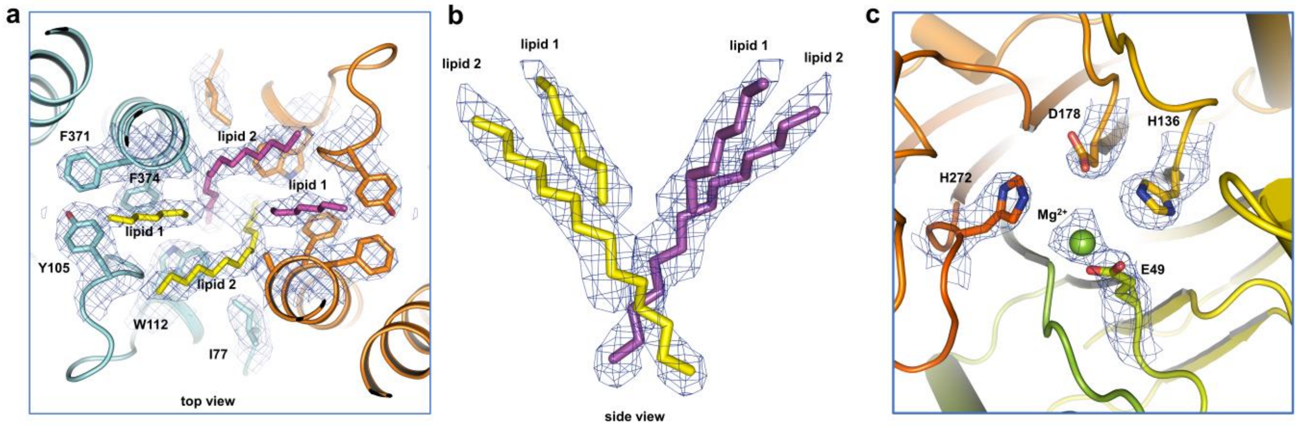

Supplementary Fig.7 Representative densities of hSMPD2.

a. Top view of the lipid's densities and surrounding residues. The density maps were contoured at 8.0 σ.

b. Side view of the lipid's densities, contoured at 8.0 σ.

c. Representative density of Mg^{2+} and its surrounding residues, contoured at 8.0 σ.

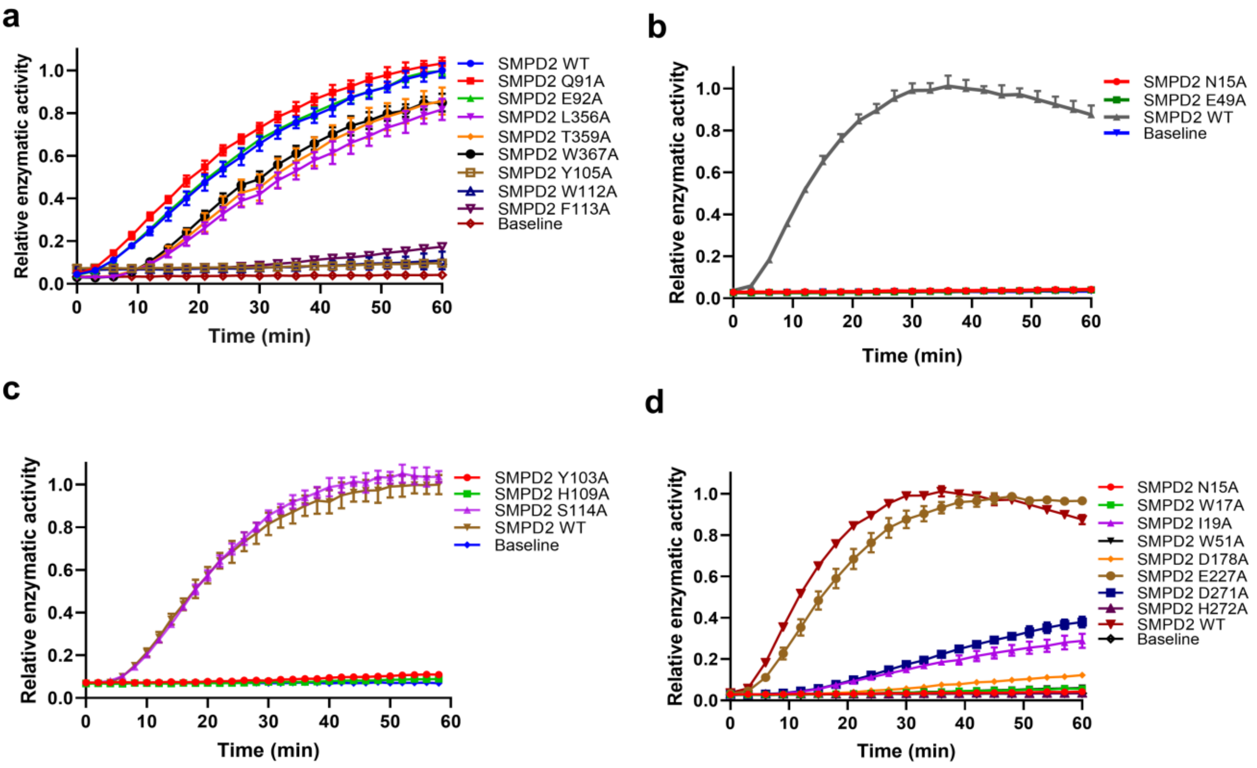

Supplementary Fig.8 Representative fluorescence traces of hSMPD2 catalysis rate.

a. Functional verification of hSMPD2 residues involved in dimeric interface. Data shown are mean +/- SEM, *n*=4 biological replicates. Source data are provided as a Source Data file.

b. Functional verification of hSMPD2 residues involved in Mg^{2+} coordination. Data shown are mean +/- SEM, *n*=4 biological replicates. Source data are provided as a Source Data file.

c. Functional verification of hSMPD2 residues in D-K loop domain. Data shown are mean +/- SEM, *n*=4 biological replicates. Source data are provided as a Source Data file.

d. Functional verification of hSMPD2 residues involved in catalytic core. Data shown are mean +/- SEM, *n*=4 biological replicates. Source data are provided as a Source Data file.

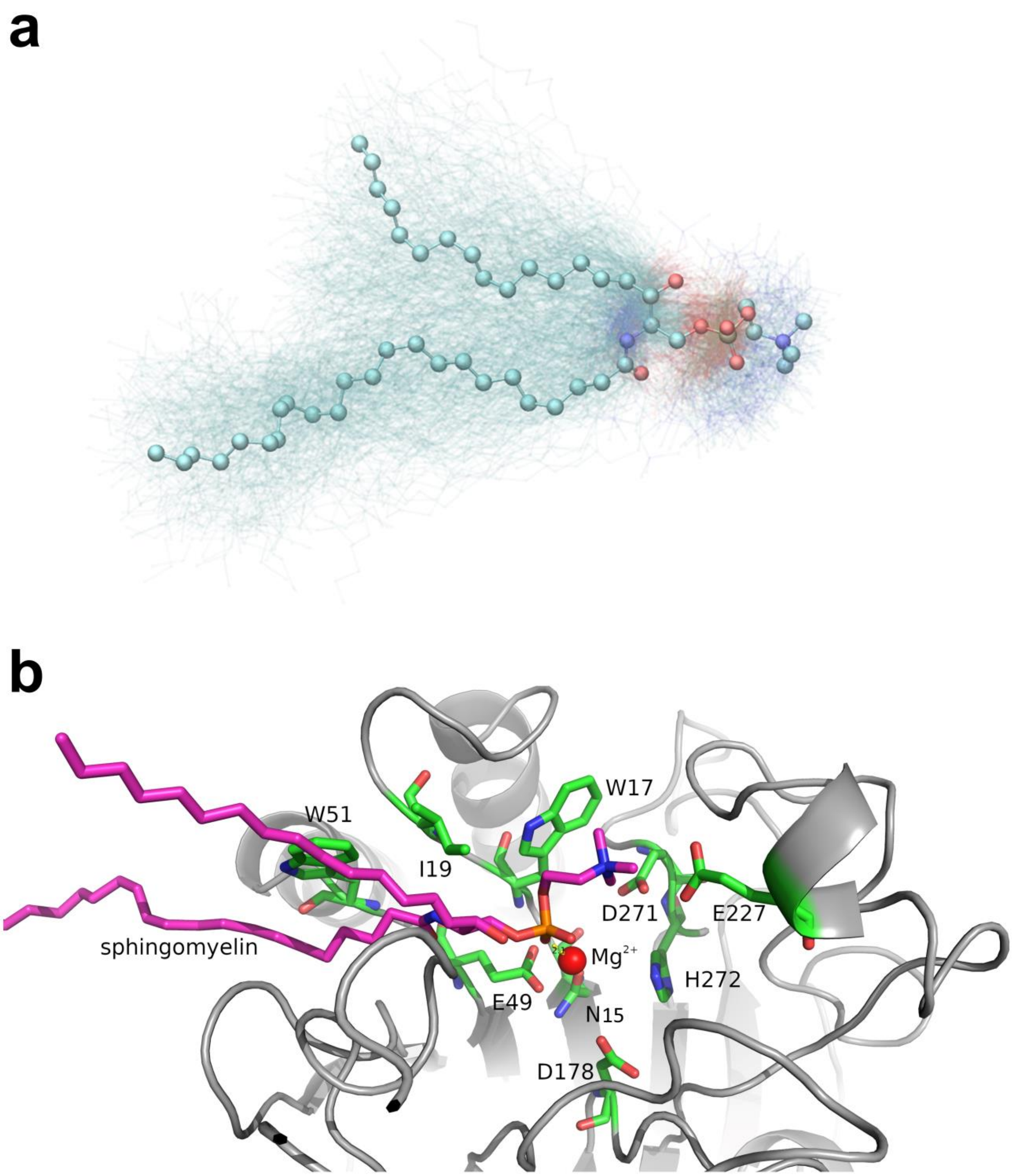

Supplementary Fig.9 Molecular docking of the sphingomyelin binding pose of hSMPD2.

a. A library of 300 conformations of sphingomyelin binding poses of hSMPD2.

b. The binding interactions of the sphingomyelin within the substrate binding pocket.

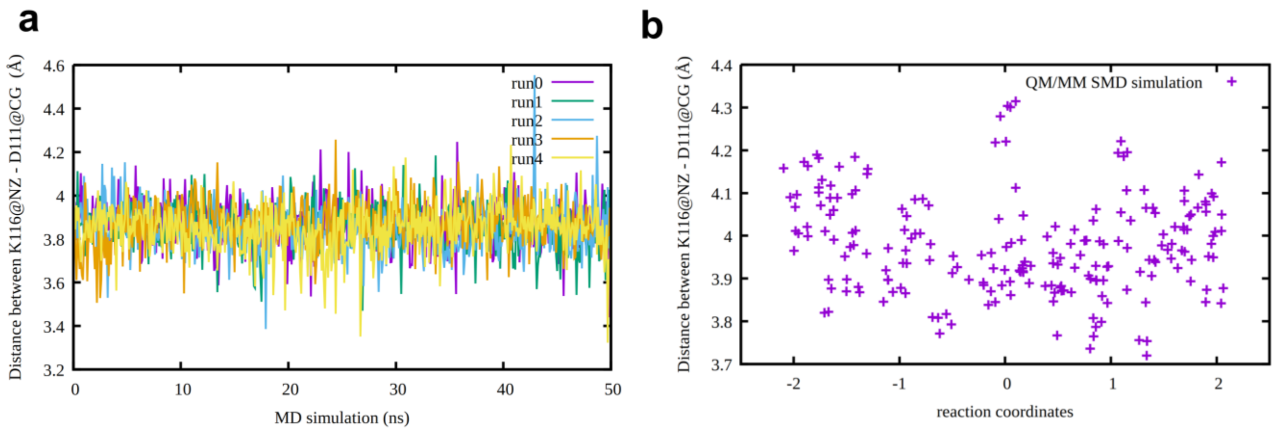

Supplementary Fig.10 The interaction between D111-K116 during simulations

a. A stable salt-bridge was formed between D111 and K116 during five independent MD trajectories.

b. The distances between K116 and D111 during QM/MM SMD simulations.

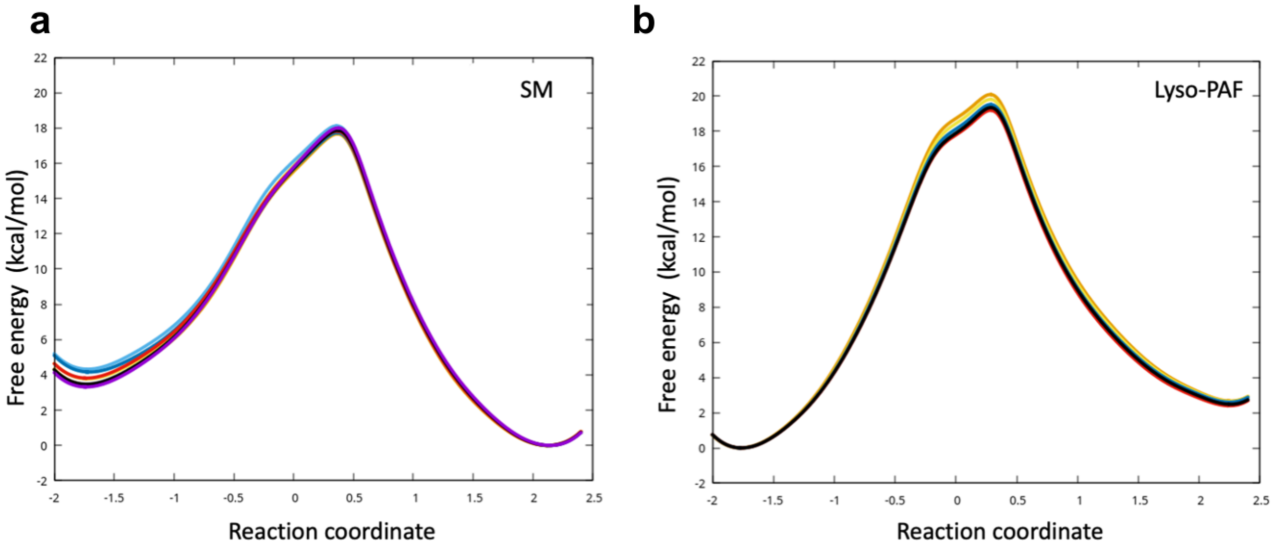

Supplementary Fig.11 Free energy convergence from QM/MM US calculations using (a)SM and (b) lyso-PAF as substrate, respectively.

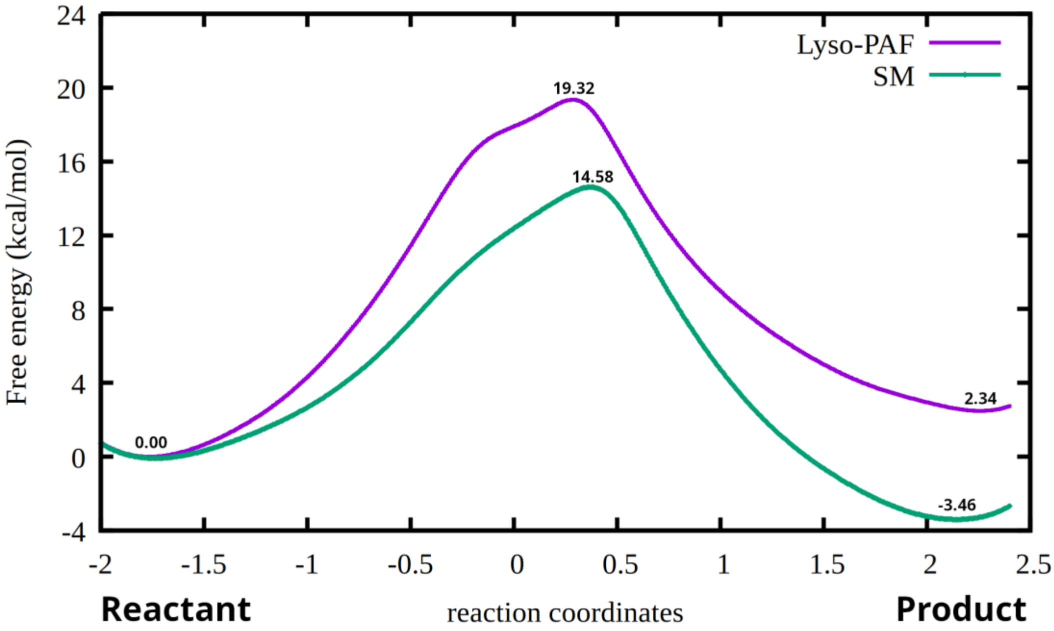

Supplementary Fig.12 Free energy profiles for our proposed catalytic mechanism using SM (green line) and lyso-PAF (purple line) as substrate, respectively.

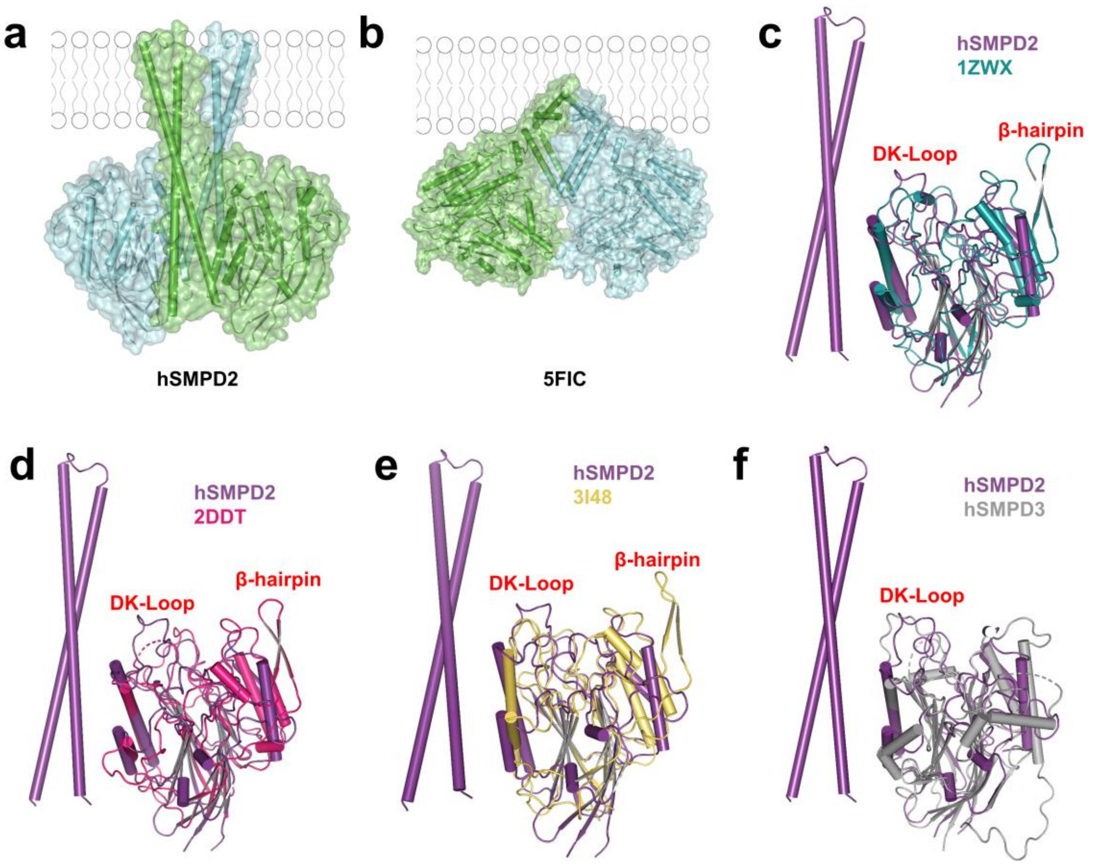

Supplementary Fig.13 Structural comparisons of nSMases.

a. Schematic representation of dimeric hSMPD2. The two protomers are indicated in green and blue.

b. Schematic representation of acid SMase (PDB code 5FIC). The two protomers are indicated in green and blue.

c. Structural comparison of monomeric hSMPD2 (purple) and Smcl (PDB code 1ZWX; green). The DK switch and β-hairpin regions are indicated in red.

d. Structural comparison of monomeric hSMPD2 (purple) and sphingomyelin phosphodiesterase from *Bacillus cereus* (PDB code 2DDT; red).

e. Structural comparison of monomeric hSMPD2 (purple) and beta toxin from *Staphylococcus aureus* (PDB code 3I48; yellow).

f. Structural comparison of monomeric hSMPD2 (purple) and hSMPD3 (PDB code 5UVG; gray).

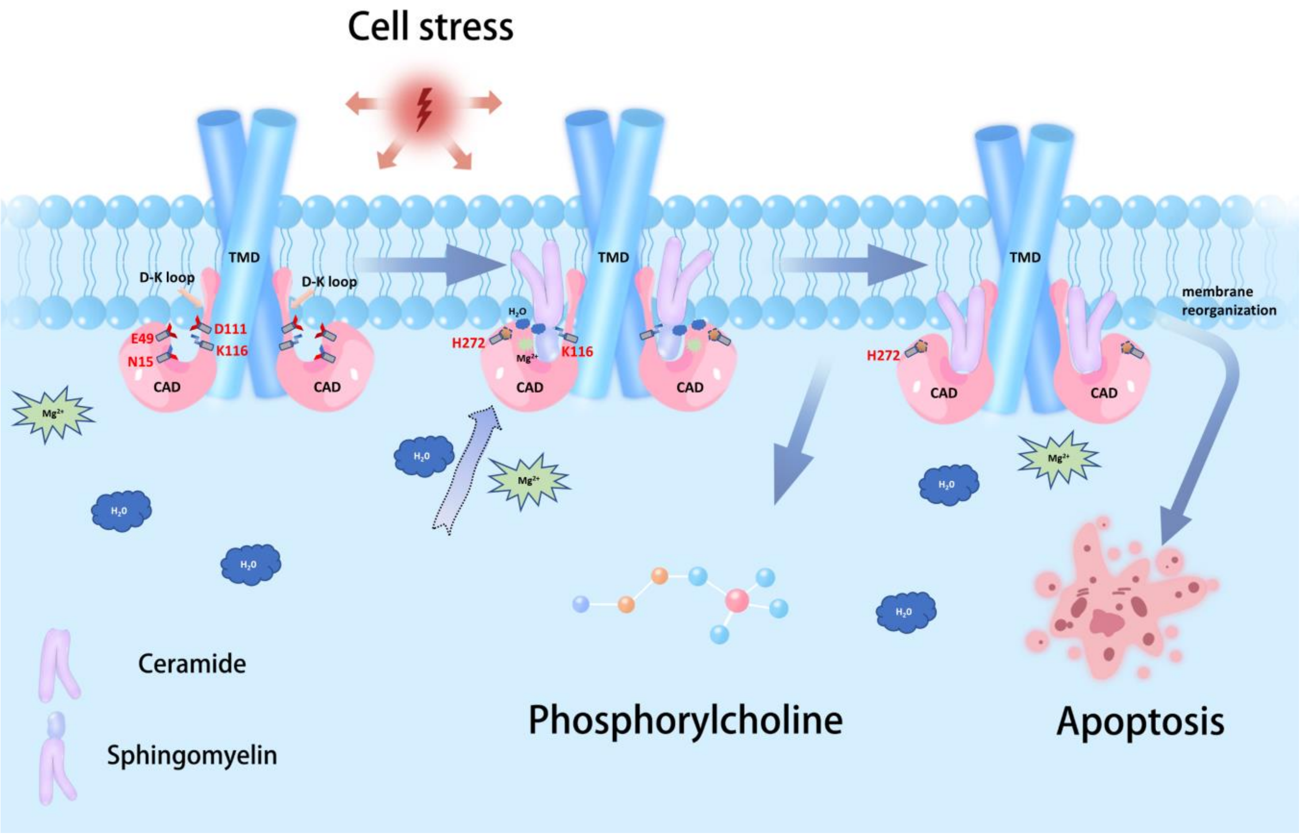

Supplementary Fig.14 Working model of the representative sphingomyelin catalytic mechanism of hSMPD2.

Under different cell stress conditions, hSMPD2 is activated and catalyzes sphingomyelin into phosphorylcholine and ceramide, which in turn trigger apoptosis.

Supplementary Table 1 | Cryo-EM data collection, refinement and validation statistics

	**hSMPD2 (EMDB-35948) (PDB-8J2F)**
**Data collection and processing**	
Magnification	105,000
Voltage (kV)	300
Electron exposure ($e^-/Å^2$)	50
Defocus range (μm)	-1.0 ~ -2.5
Pixel size (Å)	0.8374
Software	Relion-3
Symmetry imposed	C2
Initial particle images (no.)	1,334,483
Final particles images (no.)	220,338
Map resolution (Å)	3.07
FSC threshold	0.143
Local map resolution range (Å)	4.0-2.0
**Refinement**	
Initial model used (PDB code)	Alpha Fold
Model resolution (Å)	3.0/3.1
FSC threshold	0.143/0.5
Model resolution range (Å)	2.9-3.5
Map sharpening *B* factor	130.2
Model composition	
Non-hydrogen atoms	6198
Protein residues	772
Ligand	6
B factors ($Å^2$)	
Protein	63.19
Ligand	47.69
R.m.s deviations	
Bond length (Å)	0.007
Bond angles (°)	1.075
Validation	
MolProbity score	1.21
Clashscore	2.68
Poor rotamers (%)	0
Ramachandran plot	
Favored (%)	97.12
Allowed (%)	2.88
Disallowed (%)	0

Supplementary Table 2 | The composition of the bilayer (number of lipids)

	POPC	POPS	SM
**Upperleaflet**	80	80	22
**Lowerleaflet**	95	100	10

POPC = 1-palmitoyl-2-oleoyl-sn-glycero-3-phosphocholine

SM = sphingosine-phosphorylcholine;

POPS = 1-palmitoyl-2-oleoyl-sn-glycero-3-phospho-L-serine.
