# Supplementary material for: Molecular basis for the catalytic mechanism of human neutral sphingomyelinases 1 (hSMPD2)
Source: Nat Commun. 2023 Nov 27;14:7755. doi: 10.1038/s41467-023-43580-w (PMC10682184; doi:10.1038/s41467-023-43580-w)
Supplement: Supplementary file 3 — Description of Additional Supplementary Files [file 41467_2023_43580_MOESM3_ESM.pdf]

File name: Supplementary Data 1

Description: QM/MM steered MD (SMD) simulations were performed to drive the catalytic reaction in the forward direction from the reactant state to the product state by defining the reaction coordinate as the distance difference between the scissile bond and the forming bond, which further revealed that H272 acts as a general base to accept the proton and to facilitate the reaction.

File name: Supplementary Data 2

Description: MD Simulation Related Files, showing input files and final output files for SM and lyso-PAF simulations, respectively.
