# Supplementary material for: Molecular basis for the catalytic mechanism of human neutral sphingomyelinases 1 (hSMPD2)
Source: Nat Commun. 2023 Nov 27;14:7755. doi: 10.1038/s41467-023-43580-w (PMC10682184; doi:10.1038/s41467-023-43580-w)
Supplement: Supplementary file 6 — Reporting Summary [file 41467_2023_43580_MOESM6_ESM.pdf]

nature portfolio

Corresponding author(s): Maojun Yang

Last updated by author(s): Oct 20, 2023

Reporting Summary

Nature Portfolio wishes to improve the reproducibility of the work that we publish. This form provides structure for consistency and transparency in reporting. For further information on Nature Portfolio policies, see our Editorial Policies and the Editorial Policy Checklist.

Statistics

For all statistical analyses, confirm that the following items are present in the figure legend, table legend, main text, or Methods section.

n/a	Confirmed	
☐	☒	The exact sample size (*n*) for each experimental group/condition, given as a discrete number and unit of measurement
☐	☒	A statement on whether measurements were taken from distinct samples or whether the same sample was measured repeatedly
☒	☐	The statistical test(s) used AND whether they are one- or two-sided<br>*Only common tests should be described solely by name; describe more complex techniques in the Methods section.*
☒	☐	A description of all covariates tested
☒	☐	A description of any assumptions or corrections, such as tests of normality and adjustment for multiple comparisons
☐	☒	A full description of the statistical parameters including central tendency (e.g. means) or other basic estimates (e.g. regression coefficient) AND variation (e.g. standard deviation) or associated estimates of uncertainty (e.g. confidence intervals)
☒	☐	For null hypothesis testing, the test statistic (e.g. *F*, *t*, *r*) with confidence intervals, effect sizes, degrees of freedom and *P* value noted<br>*Give P values as exact values whenever suitable.*
☒	☐	For Bayesian analysis, information on the choice of priors and Markov chain Monte Carlo settings
☒	☐	For hierarchical and complex designs, identification of the appropriate level for tests and full reporting of outcomes
☒	☐	Estimates of effect sizes (e.g. Cohen's *d*, Pearson's *r*), indicating how they were calculated

Our web collection on statistics for biologists contains articles on many of the points above.

Software and code

Policy information about availability of computer code

Data collection	Cryo-EM data were collected by AutoEMation2.0
Data analysis	For cryo-EM data processing: Relion, PHENIX1.14-3374, ResMap1.1.4,<br>For cryo-EM atomic model refinement and analysis: Coot0.8.9, PHENIX1.14-3374, MolProbity (part of PHENIX package),<br>For cryo-EM figure preparation: PyMol2.1.0, Chimera1.14, ChimeraX1.25, ESPpript3<br>For assay data representation and statistical analysis: GraphPad Prism Version 8.2.1

For manuscripts utilizing custom algorithms or software that are central to the research but not yet described in published literature, software must be made available to editors and reviewers. We strongly encourage code deposition in a community repository (e.g. GitHub). See the Nature Portfolio guidelines for submitting code & software for further information.

Data

Policy information about availability of data

All manuscripts must include a data availability statement. This statement should provide the following information, where applicable:

- Accession codes, unique identifiers, or web links for publicly available datasets
- A description of any restrictions on data availability
- For clinical datasets or third party data, please ensure that the statement adheres to our policy

Data that support the findings of this study have been deposited in the Worldwide Protein Data Bank with the accession codes 8J2F. The corresponding maps have been deposited in the Electron Microscopy Data Bank with the accession codes 35948.

Field-specific reporting

Please select the one below that is the best fit for your research. If you are not sure, read the appropriate sections before making your selection.

- [x] Life sciences
- [ ] Behavioural & social sciences
- [ ] Ecological, evolutionary & environmental sciences

For a reference copy of the document with all sections, see nature.com/documents/nr-reporting-summary-flat.pdf

Life sciences study design

All studies must disclose on these points even when the disclosure is negative.

Sample size	No sample-size calculation was performed.
Data exclusions	Only bad cryo-EM micrographs or particles were excluded to get a high resolution map.
Replication	All attempts at replication were successful.
Randomization	Randomization is not relevant to the majority of experiments of this study, because protein samples are not required to be allocated into experimental groups in the biochemical studies. Randomization was used only in cryo-EM image processing and structure refinement.
Blinding	The researchers were not blinded to group allocation, because samples must be grouped in mutagenesis analysis (WT vs mutations for expression and transfection) during the experimental performances.

Reporting for specific materials, systems and methods

We require information from authors about some types of materials, experimental systems and methods used in many studies. Here, indicate whether each material, system or method listed is relevant to your study. If you are not sure if a list item applies to your research, read the appropriate section before selecting a response.

Materials & experimental systems

n/a	Involved in the study
[ ]	[x] Antibodies
[ ]	[x] Eukaryotic cell lines
[x]	[ ] Palaeontology and archaeology
[x]	[ ] Animals and other organisms
[x]	[ ] Human research participants
[x]	[ ] Clinical data
[x]	[ ] Dual use research of concern

Methods

n/a	Involved in the study
[x]	[ ] ChIP-seq
[x]	[ ] Flow cytometry
[x]	[ ] MRI-based neuroimaging

Antibodies

Antibodies used	Primary antibodies used in this study with supplier, catalog number and the used dilution.<br>anti-Strep-Tag monoclonal antibody; catalogue number BE2076, Bioeasytech, (Western Blotting, Mouse, 1:3000)<br>anti-Flag-Tag monoclonal antibody; catalogue number BE7003, Bioeasytech, (Western Blotting, Mouse, 1:3000)<br>anti-Flag-Tag monoclonal antibody; catalogue number F3040, Merck, (immunofluorescence, Mouse, 1:500)<br>anti-Sec61B monoclonal antibody; catalogue number 14648, Cell Signaling Technology, (immunofluorescence, Rabbit, 1:300)<br>anti-Giantin antibody; catalogue number ab80864, Abcam, (immunofluorescence, Rabbit, 1:300)<br>Wheat Germ Agglutinin (WGA), catalogue number W32466, Invitrogen, (immunofluorescence, 1:300)
Validation	Concerning antibody specificity, we refer to the supplier's websites and datasheets to find statements on specificity and dilution for

the use of the antibodies:
anti-Strep-Tag monoclonal antibody; http://www.bioeasytech.com/product/2442.html?goods_id=4427
anti-Flag-Tag monoclonal antibody; http://www.bioeasytech.com/product/3393.html?goods_id=6568
anti-Flag-Tag monoclonal antibody; https://www.sigmaaldrich.cn/CN/zh/product/sigma/f3040
anti-Sec61B monoclonal antibody; https://www.cellsignal.com/products/primary-antibodies/sec61b-d5q1w-rabbit-mab/14648
anti-Giantin antibody; https://www.abcam.cn/products/primary-antibodies/giantin-antibody-golgi-marker-ab80864.html
Wheat Germ Agglutinin (WGA), https://www.thermofisher.cn/order/catalog/product/W32466

Eukaryotic cell lines

Policy information about cell lines

Cell line source(s)	FreeStyle™ 293-F Cells were from Invitrogen, Catalog number, R79007; SY5Y cells were from ATCC, Catalog number, CRL-2266.
Authentication	SY5Y cells was authenticated by STR profiling. FreeStyle™ 293-F Cells for protein expression were not authenticated.
Mycoplasma contamination	The cell line tested was negative for mycoplasma contamination.
Commonly misidentified lines (See ICLAC register)	No commonly misidentified cell lines were used.
